# Validation of content and structure of the Return-to-work assessment for post-stroke survivors

**DOI:** 10.4102/sajp.v78i1.1790

**Published:** 2022-10-27

**Authors:** Peter O. Ibikunle, Anthea Rhoda, Mario R. Smith, Ushotanefe Useh

**Affiliations:** 1 Department of Physiotherapy, Faculty of Community and Health Sciences, University of the Western Cape, Cape Town, South Africa; 2 Department of Medical Rehabilitation, Faculty of Health Sciences and Technology, Nnamdi Azikiwe University, Nnewi, Nigeria; 3 Department of Lifestyle Diseases, Faculty of Health, North-West University, Mafikeng, South Africa; 4 Department of Psychology, Faculty of Community and Health Sciences, University of the Western Cape, Cape Town, South Africa

**Keywords:** disability, scale, WHO, rehabilitation, validation

## Abstract

**Background:**

Validation of an instrument consist of three main types: content, criterion and construct. Content validity needs to be determined in order for an instrument to be acceptable for use, validity establishes the fact that an instrument measures exactly what it proposes to measure. The Return-to-work assessment scale (RAS) was developed to measure three aspects of return to work: (Personal factors and/or issues, work issues and contextual factors) in 2021.

**Objective:**

To report on the processes followed in establishing the face and content validity of the RAS.

**Method:**

Twenty participants took part in our study, they were selected purposively and conveniently from a pool of professionals and post stroke survivors. The Delphi survey technique was used to arrive at consensus and professional opinion on the items included in the RAS. Consensus was sought on the items, domains and subdomains included in the RAS that was used to assess return-to-work after a stroke. Our study was concluded after the third round.

**Result:**

One item was remove out of the original 86, three (3) domains made up of eleven (11) subdomains were retained. The RAS had consensus of 100% after three rounds of scrutiny for all items.

**Conclusion:**

The RAS was found to be valid, thereby establishing its face and content validity.

**Clinical implication:**

The RAS is valid and was recommended for psychometric testing which was the next stage after face and content validity.

## Introduction

Work is not only *a job* or paid employment but also unpaid or voluntary work, education and training, family responsibilities and caring (Balasooriya-Smeekens et al. 2016; Waddell & Burton 2006). Work usually involves commitment over time and a need to labour or exert oneself. In addition, it connotes the application of physical or mental effort, skills, knowledge or other personal resources (Balasooriya-Smeekens et al. 2016; Ibikunle et al. 2021:9b; Waddell & Burton 2006).

Stroke impacts a survivor’s ability to return to work (RTW), thereby affecting participation in community activities, especially individuals who are still in the working age range (Ibikunle et al. 2021b:9; Saeki, 2000), which creates activity limitation and participation restriction.

Return to work is essential, as through the investments gained from income, it contributes to life satisfaction and social identity. Return to work has been investigated empirically; however, two core concerns have been raised: (1) the need for a comprehensive and multiperspective measurement of the factors that predict RTW and (2) the cited differences in issues related to stroke between developed and developing countries (Ibikunle et al. 2021b:7; Soklaridis, Ammendolia & Cassidy 2010).

Content validity refers to the degree in which the instrument content sufficiently reflects the construct that is being measured (Polit 2015). It evaluates to what extent the items sampled represent in a content domain (Polit & Beck 2011). Functional scales that focus on the measurement of the impact of disease on performance of everyday tasks are now commonly employed by clinicians and researchers (Ibikunle et al. 2021b:5; Muller, Roder & Greennough 2006; Sinha, Nijhawan & Grover 2014). They are usually classified as generic or disease specific. General health outcome measures are intended towards recapitulating or summarising details of the outcomes of most health conditions among patients and populations, while disease-specific outcome measures evaluate the impact of specific health conditions on the functional status of patients (Ibikunle et al. 2021b:5; Kampstra et al. 2018; Partrick 1990). These disease-specific outcome measures are observed to be more responsive to the target population when compared to generic measures (Davidson & Keating 2002; Ibikunle et al. 2021a; Kampstra et al. 2018; Muller et al. 2006).

The return-to-work assessment scale (RAS) was developed by Ibikunle et al. (2021a) as a health-specific scale for the purpose of measuring return to work among post-stroke survivors. The International Classification of Functioning, Disability and Health (ICF) and the Flag model were used for the conceptual mapping, and the theoretical framework adopted for the measure was the modified C-OAR-SE theory (Ibikunle et al. 2021a). C-OAR-SE is an acronym for the six aspects of the theory: ‘C’ stands for construct definition, ‘OAR’ for object representation, attribute classification and rater entity identification, and ‘SE’ for selection of item type and answer scale, as well as enumeration and scoring rules (Diamantopoulos 2005; Ibikunle et al. 2021a; Rossiter 2011a).

The three phases of development of the RAS as described by Ibikunle et al. (2021a) are (1) construct development (initial item generation), (2) face and content validation of the instrument and (3) psychometric testing. Return to work was conceptualised into three views: (1) personal factors that include grooming, independence, psychological and emotional balance; (2) work-related issues such as mobility, employees’ and employers’ attitudes and infrastructures and (3) contextual issues that were limited to support from family, relations, coworkers and society (company or country labour policies were not considered in our study). These three issues gave theoretical definition to RTW, each aspect answering to the capacity of the post-stroke survivor to predict returning to work, which brings independence, self-esteem and improved quality of life – the core values that can be achieved through rehabilitation. The conceptual framework was the ICF saddled with the flag model in developing the instrument using the six stages of thematic analysis after obtaining the in-depth interviews from the participants.

Many outcome measures used for the measuring of various variables among stroke patients include the Gross Motor Rivermead Assessment Scale by Lincoln and Gladman (1962), the Barthel Index by Mahoney and Barthel (1965) and the Nottingham Extended Activities of Daily Living by Finch et al. (2002), to mention but a few. Developing a health-specific outcome measure to assess return to work among stroke survivors will help to assess their readiness, as well as to monitor the RTW stages. With no means of evaluating return to work among post-stroke survivors, they will not be accepted back into their formal employment, which could affect their self-esteem, confidence and social identity (Balasooriya-Smeekens et al. 2016; Ibikunle et al. 2021b:7). Consequently, there is a need to develop an instrument that takes into consideration the contextual issues and the viewpoints of the employers and employees, as well as other important issues peculiar to post-stroke survivors, namely psychosocial and physical issues of the clime and region. Our study, which is the second phase of the research, consists of face and content validation. In this phase, consensus was sought on the items, domains and subdomains included in the RAS, which were used to assess return to work after stroke (Ibikunle et al. 2021b:8). The Delphi survey technique was deemed appropriate, as it addressed face and content validation.

## Methods

### Procedure

Phase 1, construct development (initial item generation), utilised in-depth interviews with a set of semistructured questions (Online Appendix 1, Interview guide) to produce transcribed responses to the questions obtained from 18 participants who were the panellists. The 18 participants were invited by the author and interviewed; they included seven post-stroke survivors, five rehabilitation specialists (one occupational therapist, three physiotherapists and one occupational nurse), three caregivers and three employers of labour. Their transcribed responses (see Online Appendix 1, Interviews on return-to-work post-stroke) were analysed using the six steps of the thematic analysis by Braun & Clarke (2006) (familiarisation, coding, generating theme, reviewing theme, defining or naming and writing up), thereby arriving at five themes which emerged from using the ATLAS.ti 7.5.0 software. The transcribed interviews were converted to PDF and uploaded into the qualitative analysis tool. Common concepts were identified, from which codes and quotations were derived, and later themes emerged which were extracted and transcribed. The five themes obtained were (1) impairment and functional limitations resulting from stroke; (2) cognitive and psychological limitations resulting from stroke; (3) barriers to RTW post-stroke; (4) facilitators of RTW post-stroke and (5) stroke as a social responsibility. The five themes were further compressed by the author and the study supervisors, who are experts in instrument development, in a focus group discussion (virtual) which produced 3 domains, 11 subdomains and 85 items. The development of a draft document, referred to as the RAS, was the first phase of the research.

The next phase, referred to as phase 2, utilised the Delphi survey technique to validate the content (items) of the scale. The Delphi technique is a useful research tool that can be used to obtain consensus from a chosen group (Gupta & Clarke 1996). The Delphi technique was selected to obtain expert opinion, as it allows for wide consultation while eliminating geographical constraints (Gupta & Clarke 1996). Experts in the field of rehabilitation were selected from academics and healthcare practitioners, as well as acute post-stroke survivors who had recovered and had returned to work at the time of this survey. Twenty-five experts and stakeholders were approached to participate in the study, being 10 experts from South Africa, 10 from Nigeria and 5 post-stroke survivors from Nigeria. However, of the 25 approached, only 20 of these experts agreed to participate in our study. Four professionals from South Africa did not respond, and one declined to participate because of inadequacy of required knowledge. The professionals were to have at least between 5 and 10 years of clinical experience in neurological rehabilitation. The group which participated included 10 rehabilitation experts (eight physiotherapists and 2 occupational therapists), 3 psychologists, 2 employers and 5 post-stroke survivors. The employers had at least between 5 and 10 years’ experience of recruiting workers for employment. The psychometrists were experts in the development of instruments and psychometrics. The psychologists, who had health psychometrics backgrounds, assisted with the development and finalisation of a scoring system for the newly developed instrument. Only two African countries were involved, South Africa and Nigeria. This article is the product of a thesis supervised from South Africa and carried out in Nigeria; in our study, contextual factors did not include country, company laws or policies, and this was not part of the scope of our study. Contextual factors were limited to support from family, relations, coworkers and society.

### Data collection and stimulus prompt

The draft instrument was used as the stimulus prompt in the Delphi process. The RTW outcome measure consisted of two sections. Section A involved general sociodemographic issues, while section B contained the three main domains. In section A, the participants were requested, on the Delphi form, to state how relevant each question in the general section was to their respective subheadings. They were requested to answer yes or no – yes, if relevant, and no, if not. In addition, they were invited to suggest any other question that they deemed relevant, if not already included in the scale.

### Delphi survey technique – Rounds 1 – 3

A three-round e-Delphi technique was employed. The objectives of these rounds were to establish face and content validity, obtain consensus regarding items to be included in the outcome measure, assess return to work among post-stoke survivors and develop the overall scoring system. The draft measure was revised during the Delphi rounds. The revised measure was used in phase 3 to establish the psychometric properties.

#### Delphi technique round 1

This was the first round; the preliminary instrument which was developed was e-mailed to experts and participants for three weeks, with e-mails sent at intervals to monitor compliance to request.

#### Delphi technique round 2

Item reduction was the purpose of this second stage. Any item that did not obtain a consensus of 100% from experts and participants was resent for the purpose of complete consensus.

#### Delphi technique round 3

In this third and last round, the third draft with all corrections effected was sent to the experts and participants. The health psychologists were involved from the start, especially regarding issues related to the scoring system. All participants accepted the scoring system as presented by the authors and perceived no need for any change. The new instrument had to cover the domains that constitute return to work among post-stroke survivors. The level of consensus reached on the suitability and appropriateness of each item included in the scale at the end of the Delphi study was 100%.

There is no consistent reference available to determine the standard level of consensus. The three rounds of the Delphi technique were hence used to facilitate reaching consensus; the percentage set for consensus was 100%. (Ibikunle et al. 2021b:48)

#### Time requirements

Two weeks were given as the minimum time for the experts and participants to respond; we adopted 6 weeks as the maximum time for each round of the Delphi process.

### Ethical considerations

Ethics approval and project registration were sought and obtained from the Senate Research Committee of the University of the Western Cape (reference number: 15/2/20). In Nigeria, ethics approval was also sought from the Faculty of Health Sciences and Technology Ethics Committee of Nnamdi Azikiwe University (reference number: ERC/FHST/NAU/2018/028).

## Results

### Sociodemographic distribution of participants

Table 1 reveals 20 participants who consented: 15 from Nigeria and 5 from South Africa, specifically the University of the Western Cape (4 participants) and North-West University (1 participant). The response rate was 80%.

**TABLE 1:** Demographic characteristics of the panel of participants (*n* = 20).

ID	Age	Sex	Highest qualification	Current occupation of participants	Years of experience	Role in stroke rehabilitation
1.	50	Male	Higher diploma	Businessman	20	Post-stroke survivor
2.	45	Male	MBA	Banker	15	Post-stroke survivor
3.	55	Male	Diploma	Civil servant	20	Post-stroke survivor
4.	59	Male	Diploma	Businessman	30	Post-stroke survivor
5.	50	Female	BSc	Nurse	30	Post-stroke survivor
6.	39	Male	PhD	Health psychologist	8	Health psychologist involved in rehabilitation
7.	50	Male	PhD	Clinical psychologist	25	Academic, lecturer
8.	30	Male	BSc	Occupational therapist	5	Clinician
9.	35	Male	BMR(PT)	Neurophysiotherapist	18	Clinician
10.	39	Male	BMR(PT)	Neurophysiotherapist	18	Clinician
11.	50	Male	PhD	Applied social psychologist	20	Professor
12.	48	Female	PhD	Physiotherapist	29	Senior lecturer and academic
13.	50	Male	MBA	Human resources manager	20	Human resources manager
14.	38	Male	MBA	Bank manager	13	Employer
15.	38	Male	BMR(PT)	Physiotherapist	10	Clinician
16.	50	Male	PhD	Physiotherapist	29	Senior lecturer, physiotherapist
17.	50	Female	PhD	Neurophysiotherapist	20	Professor
18.	43	Female	PhD	Physiotherapist	18	Senior lecturer
19.	50	Male	PhD	Physiotherapist and exercise physiologist	30	Professor
20.	43	Male	PhD	Occupational therapist	19	Professor

B.sc, Bachelor of science; BMR (PT), Bachelor of medical rehabilitation (Physiotherapy); MBA, Masters of business Administration; PhD, Doctor of philosophy.

### Results of round 1

#### Section A of the return-to-work scale

This section is made up of five subsections, including demographic information; type, area and severity; impairments or defects; post-stroke management and nature of employment (see Online Appendix 1, Return-to-work assessment scale).

#### Section B of the return-to-work scale

This section contains the 3 domains, 11 subdomains and 86 items that were endorsed to assess return to work among post-stroke survivors. These, along with their scoring patterns, are presented in Tables 2–7.

**TABLE 2:** Consensus and comments on Delphi study on section A.

	Subsections	Consensus (%)	Number of responses	Comment
1.	Demographic information	95	19/20	This subsection was made up of two items: gender and race. One expert suggested that age should be included.
2.	Types, area and severity	100	20/20	Four items were included in this subsection, namely side affected by stroke, location or area of brain affected, grading of the stroke, date of onset of stroke.
3.	Impairments or defects	90	18/20	This subsection contains four items. The initial question was, ‘Do you have any of the following? Paralysis? Which side? Which limbs are affected? Speech defects? Cognitive defects?’ Suggestions were made to break down the questions and simplify cognitive defects.
4.	Post-stroke management	90	18/20	Six items were included in this subsection, namely hospitalisation, rehabilitation services, intensity, frequency, length and comorbid diagnoses. There was a suggestion to break down the level of hospitalisation for improved understanding.
5.	Nature of employment	80	16/20	This subsection comprised 17 items, including the nature and period of employment, health policy in place of employment, type of employment, as well as level of communication and interaction in place of employment. Suggestions were made to redefine the type of employment.

**TABLE 3:** Items included in domain 1.

Items	Number of responses (*n*)	Level of consensus (%)	Comments of experts
**1.1: Instrumental activities of daily living**			
1. I can bathe myself.	20/20	100	None
2. I can groom myself (shave or put on make-up).	20/20	100	None
3. I can dress myself.	20/20	100	None
4. I can feed myself.	20/20	100	None
5. I can use the bathroom.	20/20	100	None
6. I can exercise bowel control.	20/20	100	None
7. I can exercise bladder control.	20/20	100	None
8. I can work unaided.	20/20	100	None
9. I can use public transport.	20/20	100	None
10. I can drive myself.	20/20	100	None
11. I can travel from home to required destination.	20/20	100	None
**1.2: Cognition**			
1. Loss of interest in activities.	20/20	100	None
2. Difficulty in remembering events.	20/20	100	None
3. Difficulty in remembering people.	20/20	100	None
4. Difficulty in articulating words.	20/20	100	None
5. Talking excessively.	20/20	100	None
6. Restless and agitated.	20/20	100	None
7. Difficulty in remembering places.	20/20	100	None
**Psychosocial factors**			
8. Becoming sad, depressed and unnecessarily emotional.	20/20	100	None
9. Becoming anxious and worried.	20/20	100	None
10. Becoming angry.	20/20	100	None
11. Becoming hostile.	20/20	100	None
**1.3: Communication (Psychosocial factors)**			
1. I can follow discussions.	20/20	100	None
2. I can articulate or express my thoughts clearly to others.	20/20	100	None
3. I can interact with others without difficulty.	20/20	100	None
**1.4: Coping**			
1. I can work with instruments in my former workstation.	20/20	100	None
2. I can feel objects when handling them.	20/20	100	None
3. I can do my normal duties.	20/20	100	None
4. I can work at full capacity.	20/20	100	None
5. I can withstand the pressure and stress of my former duties.	20/20	100	None
6. I can withstand the rational challenges of my job.	20/20	100	None
7. I can withstand the expressive challenges of my former duties.	20/20	100	None
**1.5: Motivation (My motivation for returning to work is…)**			
1. Fear of impact on career development.	20/20	100	None
2. Fear of loss of employment.	20/20	100	None
3. Financial.	20/20	100	None
4. Social isolation.	20/20	100	None
5. Negative impact of absence on work.	20/20	100	None
6. Negative impact of absence on perceptions of others.	20/20	100	None
7. Negative impact of absence on my mood.	20/20	100	None
8. Improved physical health.	20/20	100	None
9. Improved participation.	20/20	100	None
10. Improved ability to function independently.	20/20	100	None
11. Concerns about being perceived as disabled.	20/20	100	None

**TABLE 4:** Items included in domain 2.

Item	No. of responses (*n*)	Level of consensus (%)	Comments of experts
**Do you agree or disagree with the following statements? (2.1: Employees’ motivation)**			
1. I am recognised by my employer as important at work, irrespective of my disability.	20/20	100	None
2. There are opportunities for personal growth at work, irrespective of my disability.	20/20	100	None
3. I will get promoted when due, irrespective of my disability.	20/20	100	None
4. I feel in control and empowered as I discharge my duties, irrespective of my disability.	19/20	95	Replace ‘Discharge’ with ‘Perform’
5. I feel secure about my job and position, irrespective of my disability.	20/20	100	None
6. I am happy with my work, and I enjoy doing it irrespective of my disability.	20/20	100	None
7. I achieve my set goals at work, irrespective of my disability.	20/20	100	None
8. I have the opportunity to organise my approach to work, irrespective of my disability.	20/20	100	None
**Do you agree or disagree with these statements? (2.2: Reasonable accommodation)**			
1. I don’t need a staircase.	20/20	100	None
2. I don’t need modifications to the staircase.	20/20	100	None
3. I don’t need an elevator to ascend to my office.	20/20	100	None
4. I don’t need access to a bathroom close to my office.	20/20	100	None
5. I don’t need a change of job description.	20/20	100	None
6. I don’t need a shift of duty to enable me to cope.	20/20	100	None
7. I can only work normal hours despite my disability.	20/20	100	None
8. I can comfortably work from home and still meet my quota.	20/20	100	None
**Do you agree or disagree with these statements? (2.3: Employers motivation)**			
1. My employer will retain me irrespective of my disability if I return to work.	20/20	100	None
2. My employer will transfer me to another unit if I cannot perform my formal duties.	20/20	100	None
3. My employer will not sack me if I cannot perform my formal duties.	20/20	100	None
4. My employer takes cordial relationship with colleagues seriously.	20/20	100	None
5. My employer does not prioritise cosmetics and physical appearance.	19/20	95	Remove this item. It looks redundant.
6. My employer is willing to give fewer duties if I cannot perform my previous duties.	20/20	100	None
7. My employers is emphatic and sympathetic with me because of my disability.	20/20	100	None
8. My employer does not think less of me because of my disability.	20/20	100	None

**TABLE 5:** Items included in domain 3.

Item	No. of responses (*n*)	Level of consensus (%)	Comments of experts
**Do you agree or disagree with these statements? (3.1: Social transport)**			
1. It’s really easy for me to talk about my problems with my family and friends.	20/20	100	None
2. My spouse and children are really very supportive during difficult times.	20/20	100	None
3. My family and extended family assist me when making difficult decisions.	20/20	100	None
4. Sharing my pains and joy with my spouse and children gives me comfort and relief.	20/20	100	None
5. Sharing my pains and joys with my coworkers, friends and neighbours brings relief to me.	20/20	100	None
6. I get moral and emotional help from my spouse and children.	20/20	100	None
7. I get help from my family and friends when making important decisions that affects my work and health.	20/20	100	None
8. I get enough assistance from people around me whenever I need help.	20/20	100	None
**Do you agree or disagree with the following statements? (3.2: Local transport)**			
1. I don’t need someone to accompany me when going outdoor because of my disability.	20/20	100	None
2. My condition allows me to board, ride or disembark from a public mode of transportation (cars, bus, train).	20/20	100	None
3. I can drive a motorcycle or car to work without assistance.	20/20	100	None
4. My condition does not prevent me from travelling to my work or to disembark at my destination.	20/20	100	None
**Do you agree or disagree with the following statements? (3.3: Attitude of communities)**			
1. I would not be asked to stay away from work, religious and social groups.	20/20	100	None
2. I would not be avoided by the community members because of my condition.	20/20	100	None
3. My condition doesn’t make people to despise me and think less of me.	20/20	100	None
4. My condition doesn’t cause me shame and embarrassment in my community.	20/20	100	None
5. People won’t avoid me because of my condition.	20/20	100	None
6. Returning to work and getting a new job is not difficult.	20/20	100	None
7. My neighbours, friends, colleague and others show love to me despite my condition.	20/20	100	None

**TABLE 6:** Consensus and comments on Delphi study on section A.

Number	Subsections	Consensus (%)	Number of responses	Comment
1.	Demographic information	100	20/20	The consensus reached 100%, after age was included, resulting in three items.
2.	Types, area and severity	100	20/20	Consensus already reached in round 1.
3.	Impairments or defects	100	20/20	Suggestions were made to break down the questions and simplify defects. The suggestion was implemented by breaking defects down to speech and walking impediments, and after this inclusion, consensus reached 100%.
4.	Post-stroke management	100	20/20	There was a suggestion to break down the level of hospitalisation for improved understanding. The suggestion was implemented by including level and length in hospitalisation, after which consensus level reached 100%.
5.	Nature of employment	100	20/20	Suggestions were made to redefine the type of employment. The suggestion was implemented by elaborating employment into temporary, casual, contract or permanent, and the consensus level reached 100%.

**TABLE 7:** Items included in subdomains 2.1 and 2.3.

Item	Number of responses (*n*)	Level of consensus (%)	Comments of experts
**Do you agree or disagree with the following statements?**			
1. I am recognised by my employer as important at work, irrespective of my disability.	20/20	100	None
2. There are opportunities for personal growth at work, irrespective of my disability.	20/20	100	None
3. I will get promoted when due, irrespective of my disability.	20/20	100	None
4. I feel in control and empowered as I perform my duties irrespective of my disability.	20/20	100	None
5. I feel secure about my job and position, irrespective of my disability.	20/20	100	None
6. I am happy with my work and I enjoy doing it, irrespective of my disability.	20/20	100	None
7. I achieve my set goals at work, irrespective of my disability.	20/20	100	None
8. I have the opportunity to organise my approach to work, irrespective of my disability.	20/20	100	None
**Do you agree or disagree with these statements?**			
1. My employer will retain me irrespective of my disability if I return to work.	20/20	100	None
2. My employer will transfer me to another unit if I cannot perform my formal duties.	20/20	100	None
3. My employer will not sack me if I cannot perform my formal duties.	20/20	100	None
4. My employer takes cordial relationship with colleagues seriously.	20/20	100	None
5. My employer is willing to give less duties if I cannot perform my previous duties.	20/20	100	None
6. My employers is emphatic and sympathetic with me due to my disability.	20/20	100	None
7. My employer does not think less of me because of my disability.	20/20	100	None

**Domain 1: Personal:** This domain is made up of five subdomains: 1.1, instrumental activities of daily living; 1.2, cognition; 1.3, communication; 1.4, coping; and 1.5, motivation.

**Domain 2: Work:** This domain is made up of three subdomains: subdomain 2.1, employees’ motivation; subdomain 2.2, reasonable accommodation; and subdomain 2.3, employers’ motivation.

**Domain 3: Contextual factors:** This domain is made up of three subdomains: subdomain 3.1, social support; subdomain 3.2, local transport; and subdomain 3.3, attitudes of communities.

### Results of round 2

Because of the high consensus level in round 1, round 2 merely served to reach total consensus in areas where 100% agreement was not obtained. This was resent to the participants to ensure total consensus.

#### Section A of return-to-work scale

This section contains the result of section A of the RAS after round 2 of the Delphi Study, here the consensus among participants is now 100% (see Table 6).

#### Section B of return-to-work scale

This section contains the three domains of the RTW scale. Two domains reached 100% consensus from the experts and patients. However, in domain 2, subdomains 2.1 and 2.3 were resent after the changes were effected to ensure 100% consensus.

#### Domain 2: Work

This section refers to Domain 2, here final consensus and agreement was reached by the participants (see Table 7).

### Results of round 3

The third round of Delphi was conducted to achieve final consensus, which materialised when every participant agreed to all the sections, as well as the various domains, after the suggested changes were implemented. The scoring system was assessed along with the study and agreed upon by the 20 experts. A consensus of 100% was obtained in all items, domains and subdomains.

## Discussion

The aim of our study was to report the process followed in establishing the face and content validity of the RAS while adopting the Delphi survey technique. A draft document referred to as RAS was developed by Ibikunle et al. (2021a); its face and content validity were determined and its psychometric properties were also established. The scale was found to be reliable and structurally valid. The result of the face and content validity as reported here revealed a consensus of 100% for all items, domains and subdomains that made up the RAS among the participants of the Delphi survey, indicating good face and content validity. This is in agreement with the works of Glassel et al. (2011); Sullivan et al. (2012) and Yu et al. (2013), which also adopted the use of a Delphi survey study in establishing face and content validity as suitable and sufficient to declare validity of a newly developed instrument. The authors placed their emphasis on the high content validity (100%) of the items and answer scale or answer scales; these arguments and definitions posit content validity as necessary for reliability, reversing the usual psychometric argument that reliability is necessary for validity (Rossiter 2012). There are some RTW studies among stroke survivors carried out in South Africa, Nigeria, United Kingdom and United States of America; none of them set out to develop an instrument for assessing return to work for post-stroke survivors (Alaszewski et al. 2007; Black-Schaffer & Osberg 1990; Busch 2009; Gilworth et al. 2009; Kauranen et al. 2013; Lock et al. 2005; Obembe et al. 2010; Olaoye, Soeker & Rhoda 2021; Peters et al. 2012; Soeker & Olaoye 2017; Wolfenden & Grace 2009). Most of the studies focused on experiences of rehabilitated stroke survivors, predictors of RTW among stroke survivors and community reintegration among stroke survivors. These studies are very different from our study, and our’s is a novel attempt to assess return to work on a scale among stroke survivors, which has not been attempted before now. Our study highlights the vacuum or lack of an outcome measure or instrument designed specifically to measure or assess RTW in all these studies. This, however, was the gap which the authors filled. However, the work of Usten et al. (2010), WHODAS 2.0, is similar to the RAS, although the WHODAS 2.0 was developed as a generic health scale for measuring functioning and disability in accordance with the ICF items. The RAS is a health-specific instrument for post-stroke survivors. No Delphi survey was conducted in the development of the WHODAS 2.0; the extensive and rigorous international research involved in developing WHODAS 2.0 included (1) a critical review of the literature on conceptualisation and measurement of functioning and disability and of related instruments; (2) a systematic cross-cultural applicability study; and (3) a series of empirical field studies to develop and refine the instrument. This suggests the RAS is an instrument with very good face and content validity, suitable, easy to understand and easy to use both in the clinical and academic environment.

### Limitation

Country and company policies and laws were not part of the contextual factors studied in this study.

## Conclusion

It can be concluded from the result of the study that the RAS has good face and content validity.
